# Modelling human perception processes in pedestrian dynamics: a hybrid approach

**DOI:** 10.1098/rsos.160561

**Published:** 2017-03-01

**Authors:** A. Colombi, M. Scianna

**Affiliations:** Department of Mathematical Sciences, Politecnico di Torino, Corso Duca degli Abruzzi 24, 10129 Torino, Italy

**Keywords:** pedestrian dynamics, human perception, social force model, repulsive behaviour, localized versus distributed perception

## Abstract

In this paper, we present a hybrid mathematical model describing crowd dynamics. More specifically, our approach is based on the well-established Helbing-like discrete model, where each pedestrian is individually represented as a dimensionless point and set to move in order to reach a target destination, with deviations deriving from both physical and social forces. In particular, physical forces account for interpersonal collisions, whereas social components include the individual desire to remain sufficiently far from other walkers (the so-called territorial effect). In this respect, the repulsive behaviour of pedestrians is here set to be different from traditional Helbing-like methods, as it is assumed to be largely determined by how they perceive the presence and the position of neighbouring individuals, i.e. either objectively as pointwise/localized entities or subjectively as spatially distributed masses. The resulting modelling environment is then applied to specific scenarios, that first reproduce a real-world experiment, specifically designed to derive our model hypothesis. Sets of numerical realizations are also run to analyse in more details the pedestrian paths resulting from different types of perception of small groups of static individuals. Finally, analytical investigations formalize and validate from a mathematical point of view selected simulation outcomes.

## 1. Introduction

Pedestrian dynamics both in normal and in panic conditions have been addressed by a wide range of multidisciplinary approaches. Historically, pedestrian behaviour has been studied with empirical approaches, typically based on direct observation, photographs and time-lapse movies (e.g. [1–5]). These methodologies have been able to collect a large amount of data regarding a number of individual walking determinants, such as mean speeds and preferential directions, reactions to the presence of obstacles, nearby persons and/or attraction points, thereby providing an important descriptive value. However, they have not been satisfactory from a predictive point of view. In the last two decades, we have been therefore witnesses of an increasing number of works approaching walker dynamics with methods and tools deriving from applied physics, mathematics and engineering. In particular, theoretical models, and the resulting computational realizations, can be used for preliminary studies to test different design solutions for urban infrastructures, such as crowded facilities, subway or railway stations, stadia, pedestrian precincts, shopping malls or big buildings. Moreover, virtual simulations are able to highlight critical conditions in which crowd disasters may occur and suggest effective countermeasures to improve safety of mass events.

Recent theoretical approaches for crowd dynamics can be typically classified as *macroscopic* models (refer, for instance, to [6–13]), *mesoscopic/kinetics* methods [14–17] and *microscopic* models. In particular, microscopic approaches describe a crowd as a collection of isolated pedestrians: each of them is individually considered, assimilated to a point particle or a quasi-rigid disc and followed during motion. More specifically, a first subgroup of microscopic models is represented by the so-called cellular automata (CA, see for instance [18,19]), where each pedestrian behaves and moves according to a set of phenomenological rules that he/she executes depending on his/her individuality and/or as a reaction to extrapersonal stimuli (i.e. exerted by other walkers or by the surrounding environment). Another subtype of microscopic approaches includes instead the discrete models: they employ classical Newtonian laws of point mechanics, as the motion of each individual is described by an ordinary differential equation (ODE). Among the discrete approaches for pedestrian behaviour, the so-called *social force model* developed by Helbing and co-workers is surely one of the most celebrated (see [20–23] and references therein).

Regardless of specific differences, most microscopic-discrete models dealing with crowd dynamics show some significant similarities in the underlying assumptions, which have been derived from empirical observations [22,24,25]. First, walkers typically move to minimize the effort to reach their target destination [26]. In this respect, they try to cover the shortest possible distance at their desired speed (i.e. most comfortable and less energy consuming). Such a preferred speed depends on the situation, sex and age, time of the day, purpose of the trip, behaviour of surrounding individuals and so on. Perturbations from individual preferred strategy are assumed to result from their attempt to remain far enough from structural elements (i.e. walls, columns and border of streets) and from two kinds of interpersonal interactions: social and physical. On the one hand, physical interactions arise from collisions between individuals, or between an individual and a domain obstacle. On the other hand, the so-called social interactions do not have a physical source and reflect the desire of a pedestrian to maintain a sufficient distance from other walkers and/or to closely follow his/her groupmates [27]. All types of contributions in walker dynamics are typically taken into account by a superposition of forces/velocity components (according to the order of the model). A recent work from our group [28] has also introduced in a discrete Helbing-like approach the concept of pedestrian environmental awareness: each simulated walker is set to change his/her target destination according to new information learned about/from the surrounding environment (i.e. *assimilation process*). Such a model feature, supported by a proper evolution law for the pedestrian gazing direction, which can be partially uncorrelated or independent from the individual direction of movement, results in more realistic dynamics. For instance, when moving within a building, an individual is able to dynamically change exit strategy, opting for the nearest door, even if it is not the one he/she initially knew or decided to use.

Despite these recent improvements, purely discrete approaches still suffer from some limitations. Particularly engaging is the description of how single walkers react to the presence of nearby individuals. This issue in fact involves human *perception processes*, based on subjective and psychological arguments. In fact, even in no panic conditions, a pedestrian can rationally decide to approach and react to the presence of the nearby persons differently, i.e. according to his/her psychological state, their specificity (e.g. appearance and behaviour) and/or to environmental conditions (e.g. poor visibility). For instance, in [29,30], the author points out that different perceptions of the surroundings can lead walkers to react in a different way to the presence of nearby individuals. In particular, he therein introduces the concept of space extension as an indicator of the way in which a pedestrian perceives the position and the distribution of the other walkers. This aspect can influence the individual collision avoidance mechanism (i.e. the so-called *territorial effect*).

In this perspective, we here start from a real-world experiment and then incorporate in a discrete model for pedestrian dynamics (enriched by a proper description of individual gazing direction) the effect of different types of human perception, which are accounted for in the social repulsive term in a completely innovative way (i.e. not present in previous works either by Helbing and colleagues or by our research group). Such a new model ingredient is allowed by the definition of a proper *perception function*, which represents a measure of the spatial presence of an individual as filtered by the perception of another pedestrian. This way each walker can be set to perceive and react to the positions of the surrounding persons objectively or subjectively. In the former case, the walker considers the presence of nearby individuals accounting uniquely for their exact position, i.e. they result in localized obstacles. Conversely, in the latter case, the presence of surrounding persons is perceived to be distributed over a given region around their actual position. The proposed description of human perception is then shown to have a substantial impact on pedestrian repulsive behaviour, as provided by a comparison between numerical results and empirical observations and by a more detailed analysis of walker migratory determinants, i.e. paths.

After the above general presentation, the contents of the paper can now be outlined more precisely. In §2, we present and discuss the experimental test at the basis of our study. In §3, we then clarify the assumptions underlying our mathematical approach and present the model components. More specifically, we first describe each velocity contribution affecting pedestrian dynamics and then focus on the interpersonal repulsive term, whose dependence on individual perception processes is introduced and commented on. Section 4 contains a computational analysis of our method by means of exploratory case studies, which first reproduce the experimental setting and then investigate the effect of different types of perception on the behaviour of walkers approaching small groups of static individuals. A discussion on the results obtained by our approach, as well as on possible research perspective, is proposed in §5. The paper is finally equipped with an analytical appendix A, whose purpose is to formalize and justify from a mathematical point of view the results obtained in some of the previously proposed numerical realizations.

## 2. Experimental test

As already disclosed, we now describe an experimental test that revealed how different perceptions of the surrounding individuals give rise to completely different pedestrian dynamics: such empirical observations prompted us to modify the traditional Helbing-like approach in this respect.

**Figure 1.**
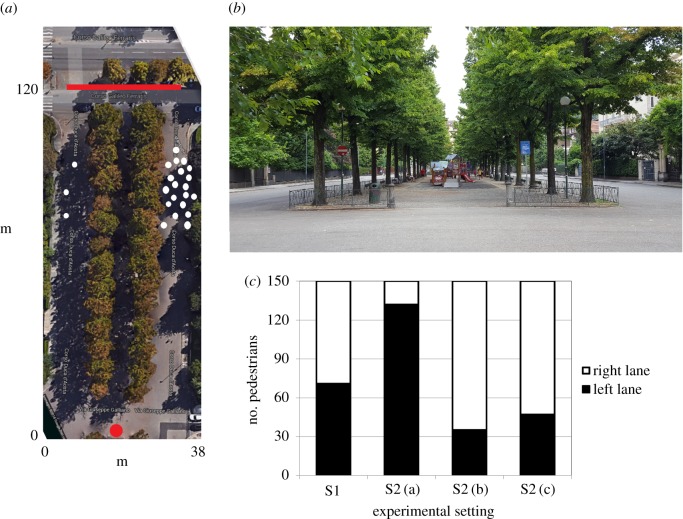
(*a*,*b*) Experimental setting: a bus stop, indicated by the red line, is located at the opposite border of a pedestrian area. The pedestrian area has in turn a non-walkable flowerbed in the middle, which identifies two equal lanes that can be alternatively used by pedestrians to reach the bus stop. A sample initial position of the test individuals is identified by the red dot. (*c*) Plot summarizing the results of the experimental test. In the absence of field individuals (setting S1), the test walkers equally opted for each of the two lanes. In the other cases, the behaviour of the test pedestrians was completely determined by how they perceived the surrounding field individuals. If the test walkers did not feel completely safe (i.e. in the case of the shabbily dressed field individuals, setting S2(b)) or were not completely aware of the behaviour of the field individuals (i.e. in the case of strolling children, setting S2(c)), they unexpectedly opted for the overcrowded right lane. In (*a*) panel, the white dots finally indicate some typical positions of the field individuals.

We studied the behaviour of individuals who aimed to reach a bus stop which extended over the entire opposite border of a pedestrian area. As illustrated in figure 1(*a*,*b*), the experimental domain of interest consisted of a pedestrian area (38 × 120 m) with a non-walkable flowerbed in the middle, which identifies two equal lanes that could be alternatively used to reach the bus stop. There were no obstacles in the middle of the lanes. We indeed investigated the individual choice of lane made by sets of single test pedestrians in the following configuration settings:

S1: absence of any other field individual;
S2: presence of three field individuals within the left lane and of 20 individuals within the right lane (figure 1*a*). In particular, such a configuration gave rise to three subcases, i.e.
  S1(a): all field individuals (both those on the left and those on the right of the flowerbed) were students similar in (normal) clothing;
  S1(b): the three field students on the left were more shabbily dressed than those on the right. This choice was made to create a substantial difference between the two groups of field individuals, in terms of appearance and possibly resulting emotional effect on the test pedestrians;
  S1(c): the three field students on the left were replaced by three children, each of them with a ball.

It is useful to notice that in all settings S2, the individuals within the left lane exactly behaved as those on the right lane, regardless of their specificity, i.e. they all stroll around their initial position. We also remark that the adjective ‘field’ is hereafter used to indicate the groups of individuals which surround and interact with the moving pedestrian of interest.

A proper statistics was then obtained for each experimental setting by taking note and counting the number of test pedestrians opting for either the left or the right lane. In this respect, to avoid possible biases, we considered for each setting a sample of 150 persons (75 males and 75 females), which covered a wide range of ages. In particular, we only considered single pedestrians: group of walkers might have been in fact affected by interpersonal influence and interactions. Furthermore, the test individuals were initially not aware of being observed, as we wanted them to behave spontaneously. A brief interview was then done once they reached the target bus stop to discuss their dynamics. Finally, the experiment was performed for four consecutive working days: in particular, on each day we focused on one experimental setting. Both the time needed to reach the bus stop and the individual paths were observed to be characterized by a substantially high variability and therefore did not represent useful quantitative determinants to further describe pedestrian behaviour (for instance, the walking times needed to cross the experimental domain ranged from 2 to 4 min, i.e. a too large interval of values). However, such aspects did not affect our analysis, as we only aimed to qualitatively characterize individual decision and phenomenology.

As reproduced in figure 1*c*, in the absence of other individuals, i.e. in setting S1, the test pedestrians equally opted for each of the two lanes. Setting S2(a) resulted instead in a significant bias towards the left lane, which was the less crowded area, as expected. Finally, in the case of configurations S2(b,c), we observed that most pedestrians took the lane on the right, see again the plot in figure 1*c*. As confirmed by the walkers themselves, the rationale of such a counterintuitive phenomenon had a psychological nature. In fact, when approaching sloppy students, the sample pedestrians had not the impression of being sufficiently safe, whereas they were not completely able to predict the behaviour of playing children. In both cases, they indeed considered the left lane as a sort of ‘dangerous/prohibited/restricted area’ which preferably had to be avoided.

The proposed experiment allowed us to draw the conclusion that the behaviour of the test pedestrians (even in a controlled quasi-normal situation) was dramatically affected by their environment, in particular by *how they perceived* the surrounding individuals. In this respect, the next step of our research study has been to check if such aspects of pedestrian dynamics could be reasonably reproduced by standard Helbing-like models or if they required the introduction of new concepts and of corresponding mathematical tools.

## 3. Mathematical model

We approach pedestrian dynamics with a discrete Helbing-like model. However, as we will see in the following, an innovative perspective will be employed to define the social term describing interpersonal repulsive interactions, which will be based on the above-explained experimental observations.

A crowd of *N* individuals, located within a bounded domain $\Omega \subset R^{2}$, which may reproduce the planimetry of a room, a square, a building, or of a park, is individually represented as a set of dimensionless points, labelled by the integer *i*∈{1,…,*N*} and identified by their actual position in space **x**_*i*_(*t*). Each pedestrian *i* is also characterized by his/her gazing direction **g**_*i*_(*t*). Therefore, the behaviour of a walker can be univocally determined by defining an evolution equation both for his/her position and for his/her visual field.

### 3.1. Pedestrian dynamics

The dynamics of a generic walker *i* can be described starting from a general second-order particle model:

3.1 $$m_{i}\frac{d^{2}{\text{x}}_{i}}{dt^{2}}(t)+\lambda_{i}\frac{d{\text{x}}_{i}}{dt}(t)={\text{F}}_{i}(t),$$

where the constants m_*i*_ and λ_*i*_ are the mass and the friction coefficient, respectively, while **F**_*i*_ denotes the sum of forces influencing individual behaviour. However, as explained in [28,31–33] and references therein, living entities (such as humans and animals) are not inanimate objects, passively dragged by external stimuli and prone to the Newtonian laws of inertia. Rather, they are intelligent individuals able to actively control their movement without undergoing inertial effects, at least for reasonable speeds, i.e. they can suddenly decide to stop and change direction of motion. These concepts allow us to neglect the inertial term in equation (3.1), i.e. to assume, in mathematical terms, that λ_*i*_>>m_*i*_ and to obtain

3.2 $$\underset{\to \;0}{\underbrace{\frac{m_{i}}{\lambda_{i}}}}\frac{d^{2}{\text{x}}_{i}}{dt^{2}}(t)+\frac{d{\text{x}}_{i}}{dt}(t)=\frac{{\text{F}}_{i}(t)}{\lambda_{i}}\Rightarrow \frac{d{\text{x}}_{i}}{dt}(t)=\frac{{\text{F}}_{i}(t)}{\lambda_{i}}=\underset{\begin{array}{c} \mathrm{pedestrian}\\ \mathrm{velocity} \end{array}}{\underbrace{{\tilde{\text{v}}}_{i}(t)}}.$$

The above equation states that the velocity of an individual, and not his/her acceleration, is proportional to the acting forces. This relation, called *overdamped force-velocity response*, is at the basis of a number of discrete/individual-based model (IBM) approaches (see [34,35] for comments) and allows selected pedestrian behaviour to be described by a direct phenomenological postulation of the velocity contributions, i.e. by a first-order model. The actual speed of a walker has also to be coherent with the specific situation (e.g. he/she is running, calm, anxious) and subjected to physical constraints. Taking these considerations into account, the equation of motion of a pedestrian *i* finally reads as

3.3 $$\frac{d{\text{x}}_{i}}{dt}(t)={\tilde{\text{v}}}_{i}(t)=\min\{{\bar{v}}_{i},|{\text{v}}_{i}(t)|\}\frac{{\text{v}}_{i}(t)}{|{\text{v}}_{i}(t)|},$$

where ${\bar{v}}_{i}\in [0,{\bar{v}}_{\max}]$ with ${\bar{v}}_{\max}$ as a maximal acceptable value for human speed. Hereafter, ${\bar{v}}_{i}$ will be constantly set equal to 1.34 m s^−1^, which is a value typically used in the literature in the case of pedestrians in a no hurry state and in quasi-normal no panicking situations (as commented in [6,36]). An analogous thresholding over pedestrian velocity has been earlier introduced in the celebrated Helbing’s model to avoid unrealistic crowd dynamics [37].

We then assume that the velocity of any pedestrian *i* results from the superposition of distinct contributions, as postulated by the well-celebrated modelling approach proposed by Helbing and co-workers [21,23,37,38] and confirmed by selected empirical evidence (cf. [39,40]). In particular, the instantaneous movement of a pedestrian is the result of his/her own strategy (developed by taking into account his/her purpose) and of the interactions with the surrounding environment and the walkers encountered along his/her motion. In this respect, the velocity components of the generic pedestrian *i* read as follows:

3.4 vi(t)=vides(t)⏟individualpstrategy+vicont(t)+virep(t)⏟interpersonalinteractions+χi(t)⏟randompfluctuations.

In the above equation, the *desired velocity* ${\text{v}}_{i}^{\mathrm{des}}$ models the walker strategy to reach its target destination. It indeed depends on personal factors (e.g. purpose of the trip) and environmental characteristics (e.g. geometry of the domain). For these reasons, ${\text{v}}_{i}^{\mathrm{des}}$ can be defined regardless of the presence of other individuals. **v**^cont^_*i*_ and **v**^rep^_*i*_ instead indicate contact and *repulsive* velocity components, respectively. They account for how the generic pedestrian *i* deviates from his/her preferred movement as a consequence of interpersonal interactions, which result from possible collisions with other individuals and from the desire to preserve a sufficient distance from them. Finally, ***χ***_*i*_ is a fluctuation term, that models possible randomness in pedestrian dynamics, as proposed for instance in [37].

#### 3.1.1. Personal pedestrian strategy of motion

The desired velocity component of a pedestrian is established according to his/her strategy to minimize the effort to reach the target destination while maintaining a reasonable distance from structural elements, e.g. the domain boundaries. In particular, according to [28], we set

3.5 $${\text{v}}_{i}^{\mathrm{des}}(t)={\text{v}}_{i}^{\mathrm{targ}}(t)+{\text{v}}_{i}^{\mathrm{wall}}(t).$$

The first velocity component in equation (3.5) is then defined by assuming that the generic pedestrian *i* prefers to cover the shortest possible path from his/her position to the target destination at his/her comfort speed. In this respect, we hypothesize that the target point/destination of interest for individual *i*, say *d*_*i*_, is a subset of the domain boundary (however, other possibilities may be taken into account, see [28]), namely ∂*Ω*_*d*_*i*__⊆∂*Ω*. ${\text{v}}_{i}^{\mathrm{des}}$ can be indeed written as follows:

3.6 $${\text{v}}_{i}^{\mathrm{targ}}(t)=-{\bar{v}}_{i}\nabla \Phi ({\text{x}}_{i}(t)),$$

where $\Phi :\Omega \mapsto R_{+}$ is a distance function, defined as

3.7 $$\Phi (\text{z}):=\min_{\text{z}\in \partial \Omega_{d_{i}}}|\text{z}-\text{x}|\;\forall \text{z}\in \Omega .$$

It can be also proven that *Φ* is the solution of the two-dimensional Eikonal equation |∇*Φ*|^2^=1, with condition *Φ*=0 on ∂*Ω*_*d*_*i*__ (see [28] and references therein).

**Figure 2.**
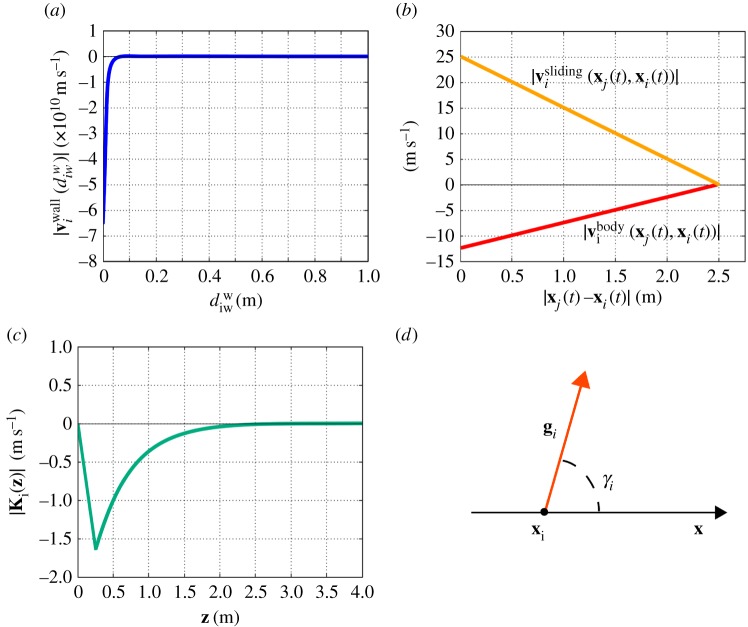
(*a*) Modulus of the velocity contribution **v**^*wall*^_*iw*_ due to the pedestrian desire to maintain a sufficient distance from a non-walkable element *w*, e.g. a structural element or a domain border, see equation (3.9). (*b*) Moduli of the contact velocity components, introduced in equation (3.10). (*c*) Modulus of the repulsive interaction kernel, as defined in equation (3.22). (*d*) Representation of the angle *γ*_*i*_ defining the gazing direction **g**_*i*_ of a generic pedestrian *i*, as described in equation (3.24).

The second velocity component in equation (3.5) instead describes the intention of the individual *i* to stay sufficiently away from (i) obstacles and structural elements within the domain; (ii) parts of the domain borders that do not represent his/her target destinations and (iii) non-walkable areas. As reproduced in figure 2*a*, such a contribution in individual dynamics can be implemented by a distance-decaying repulsive term that is active only when the pedestrian is close enough to one of them:

3.8 $${\text{v}}_{i}^{\mathrm{wall}}(t)=\sum_{w=1}^{W}{\text{v}}_{iw}^{\mathrm{wall}}(t),$$

where

3.9 $${\text{v}}_{iw}^{\mathrm{wall}}(t)=\left\{ \begin{array}{ll} -A_{i}\exp\left(\frac{\left(R_{b}-d_{iw}^{w}({\text{x}}_{i}(t))\right)}{B_{i}}\right){\text{n}}_{iw}^{w}({\text{x}}_{i}(t)), & \text{if}\;d_{iw}^{w}({\text{x}}_{i}(t))\leq L_{w};\\ 0, & \text{otherwise}; \end{array}\right.$$

where $d_{iw}^{w}$ is the distance from the actual position of pedestrian *i* to the nearest point of a non-walkable element *w* (with *w*=1,…,*W*), while ${\text{n}}_{iw}^{w}$ is the corresponding unit vector (see for instance [25,28,41]). Furthermore, *A*_*i*_ and *B*_*i*_ are constants equal to 1 m s^−1^ and 0.01 m for any individual *i*, respectively (as estimated in [28] with a proper sensitivity analysis), and *R*_b_=0.25 m is the body radius of a medium-size pedestrian (as proposed in [28,36,42,43]). Finally, *L*_w_=1 m is a reasonable threshold value above which the repulsive term from domain structural elements vanishes.

#### 3.1.2. Contact velocity

The contact velocity component reproduces the forces of sliding and pushing between two colliding persons. It has therefore a physical nature and accounts for the mass extension of each walker, which is given in the model by the already-introduced body measure *R*_b_. In particular, as plotted in figure 2*b*, the velocity of a pedestrian *i* due to contact interactions reads as follows:

3.10 $$\begin{array}{rl} {\text{v}}_{i}^{\mathrm{cont}}(t) & \;=\sum_{j\in C_{i}(t)}{\text{v}}_{ij}^{\mathrm{cont}}({\text{x}}_{i}(t),{\text{x}}_{j}(t))\\ & \;=\sum_{j\in C_{i}(t)}[{\text{v}}_{ij}^{\mathrm{body}}({\text{x}}_{i}(t),{\text{x}}_{j}(t))+{\text{v}}_{ij}^{\mathrm{sliding}}({\text{x}}_{i}(t),{\text{x}}_{j}(t))]\\ & \;=\underset{\mathrm{body}\;\mathrm{force}\;\mathrm{component}}{\underbrace{\sum_{j\in C_{i}(t)}[-C_{i}(2R_{b}-|{\text{x}}_{j}(t)-{\text{x}}_{i}(t)|){\text{n}}_{ij}({\text{x}}_{i}(t),{\text{x}}_{j}(t))]}}\\ & \;\;+\underset{\mathrm{sliding}\;\mathrm{friction}\;\mathrm{component}}{\underbrace{\sum_{j\in C_{i}(t)}[D_{i}(2R_{b}-|{\text{x}}_{j}(t)-{\text{x}}_{i}(t)|){\text{t}}_{ij}({\text{x}}_{i}(t),{\text{x}}_{j}(t))]}}, \end{array}$$

where *C*_*i*_=25 s^−1^ and *D*_*i*_=50 s^−1^, for all *i*=1,…,*N*, are constant parameters, whose values have been empirically estimated in [28] with a proper sensitivity analysis. $C_{i}$ is instead the contact neighbourhood of a walker *i*, i.e. the set of individuals colliding with *i* at the given time *t*, i.e.

3.11 $$C_{i}(t)=\{j=1,\ldots ,N:j\neq i,|{\text{x}}_{j}(t)-{\text{x}}_{i}(t)|\leq 2R_{b}\},$$

whereas the unit vectors **n**_*ij*_ and **t**_*ij*_ are, respectively, defined as

3.12 $${\text{n}}_{ij}({\text{x}}_{i}(t),{\text{x}}_{j}(t))=\frac{{\text{x}}_{j}(t)-{\text{x}}_{i}(t)}{|{\text{x}}_{j}(t)-{\text{x}}_{i}(t)|};\;{\text{t}}_{ij}({\text{x}}_{i}(t),{\text{x}}_{j}(t))={\text{n}}_{ij}({\text{x}}_{i}(t),{\text{x}}_{j}(t))\times \text{k},$$

with **k** as the unit vector perpendicular to the domain. Entering in more details, (3.11) states that interpersonal collision comes into play when a pair of pedestrian bodies is close enough. Furthermore, contact interactions are isotropic, i.e. they happen also between individuals who do not look at each other (i.e. one may collide also with an individual coming from behind). It is also useful to remark that the velocity component in (3.10) is a slight modification of the corresponding acceleration term employed in some Helbing-like models [38].

#### 3.1.3. Perception-dependent repulsion velocity

The repulsive velocity component **v**^rep^_*i*_ defines how a pedestrian intentionally deviates from his/her preferred path to keep a sufficient distance from surrounding individuals (the so-called *territorial effect*) and to avoid possible collisions. This term has indeed no physical sources, i.e. it is not dictated by interpersonal body contacts, but rather it has a genuine social/psychological nature. As confirmed by our experimental test, the repulsive behaviour of a walker is in fact affected by his/her *perception processes*: namely, repulsive interactions depend on *how* a pedestrian actually perceives and reacts to the presence and the spatial distribution of the other individuals.

In this respect, it is consistent to first assume that a pedestrian is only influenced by persons that are sufficiently near, i.e. those falling within a given region around his/her actual position. For each walker *i*, we indeed define his/her *interaction set*

3.13 $$S_{i}(t)=\left\{j=1,\ldots ,N:\;j\neq i,\;|{\text{x}}_{j}(t)-{\text{x}}_{i}(t)|\leq R_{i},\;\frac{{\text{x}}_{j}(t)-{\text{x}}_{i}(t)}{|{\text{x}}_{j}(t)-{\text{x}}_{i}(t)|}\cdot {\text{g}}_{i}(t)\geq \cos\theta_{i}\right\}.$$

**Figure 3.**
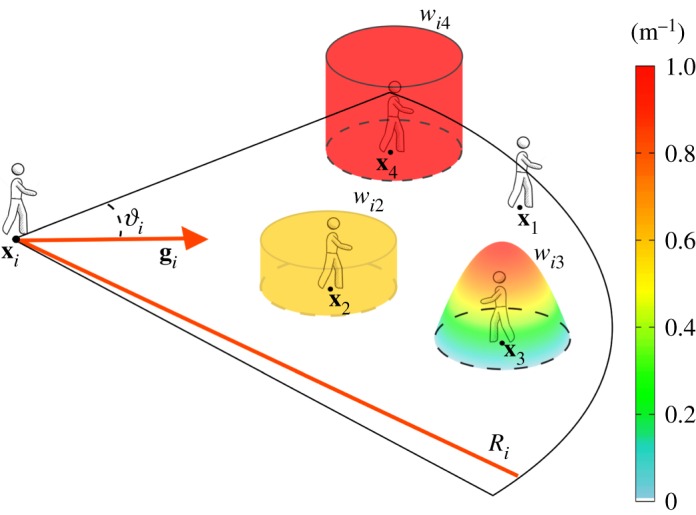
Representation of a generic pedestrian *i* and of his/her interaction set $S_{i}$, defined in equation (3.13). In particular, the walker *i* approaches the individual 1 as he/she is, i.e. a localized obstacle. The pedestrian *i* instead subjectively considers the field individuals 2, 3 and 4 as distributed entities, whose presence is psychologically perceived to be extended over the regions $I_{ij}$ (with *j*=2,3,4) and locally measured by the corresponding function *w*_*ij*_, given in equation (3.17), in equation (3.18) or in equation (3.19), respectively.

The above equation states that a pedestrian *i* accounts for only the presence of the group of individuals *j* (called again field individuals) located within a circular sector, centred at his/her position **x**_*i*_ and symmetrically enlarged from his/her gazing direction **g**_*i*_, with overall angular span 2*θ*_*i*_ and radius *R*_*i*_ (figure 3). It is worth noticing that the definition of $S_{i}$ may allow a pedestrian to react to the presence of individuals behind structural elements (for instance, walls or columns): in a first approximation, this is consistent as a walker can perceive another person also by hearing his/her voice. However, reasonable reductions of the pedestrian interaction set may be required in specific scenarios or geometries of the domain (e.g. the presence of soundproofed and/or of opaque walls), not accounted for, for the sake of simplicity, in this work. It is useful to remark that, hereafter, we assume that the extension of the interaction set is equal for all pedestrians: in particular, we fix *θ*_*i*_=1.48 *rad* (≃85^°^) and *R*_*i*_=50 m for all *i*=1,…,*N*, as done in [28,36,44].

The overall repulsive component of the velocity of a pedestrian *i* then results from the superposition of the interactions with each of his/her field individuals:

3.14 $${\text{v}}_{i}^{\mathrm{rep}}(t)=\sum_{j\in S_{i}(t)}{\text{v}}_{ij}^{\mathrm{rep}}(t)=\sum_{j\in S_{i}(t)}{\text{v}}_{ij}^{\mathrm{rep}}({\text{x}}_{i}(t),{\text{x}}_{j}(t)).$$

In (3.14), we further assume that the repulsive velocity contributions are a function of the actual position of the two interacting individuals. In this respect, as sketched in figure 3, we can distinguish two cases:

*Objective/localized perception*: Pedestrian *i* perceives the field individual *j* *objectively* as he/she is, i.e. a localized entity. In other words, pedestrian *i* regards individual *j* simply as a pointwise obstacle centred in **x**_*j*_ (see figure 3). In this perspective, the repulsion term ${\text{v}}_{ij}^{\mathrm{rep}}$ only depends on the position of the two walkers, being a function of their actual distance, i.e.
  3.15 $${\text{v}}_{ij}^{\mathrm{rep}}(t)={\text{v}}_{ij}^{\mathrm{rep}}({\text{x}}_{i}(t),{\text{x}}_{j}(t))={\text{K}}_{i}({\text{x}}_{j}(t)-{\text{x}}_{i}(t)),$$
  where ${\text{K}}_{i}:R^{2}\mapsto R^{2}$ is an interaction kernel, whose specific form and characteristics will be specified later on. The term (3.15) is the standard repulsive contribution use in Helbing-like models and does not involve subjective aspects, see [28,37,41,45].
*Subjective/distributed perceptions*: Pedestrian *i* perceives the field individual *j* *subjectively*, i.e. according to his/her appearance and behaviour or to some environmental determinants. In this respect, we here introduce an innovative concept: a pedestrian *i* can consider a field individual *j* as a distributed entity or, in other words, walker *i* accounts for the presence of individual *j* over an extended area around his/her ‘real’ location **x**_*j*_ (see again figure 3). In particular, the region of influence of field individual *j* on the dynamics of pedestrian *i* is here labelled with $I_{ij}$ and assumed to be a ball centred in **x**_*j*_ with radius *R*_*ij*_, i.e.
  3.16 $$I_{ij}=\{\xi \in \Omega :|\xi -{\text{x}}_{j}(t)|\leq R_{ij}\}.$$
  Furthermore, we define a positive Lebesgue integrable function $w_{ij}:\Omega \mapsto R_{+}$, with $\mathrm{supp}\;w_{ij}=I_{ij}$, which locally gives a measure of the presence of individual *j* as filtered by the subjective perception of pedestrian *i* (cf. figure 3). In principle, there is a wide range of options for the explicit form of *w*_*ij*_, each of them accounting for a specific situation and/or personal condition. For instance, the function *w*_*ij*_ can assume the form of a probability distribution over $I_{ij}$ (so that $\int_{I_{ij}}w_{ij}(\xi )\;d\xi =1)$, which can be uniform, i.e.
  3.17 $$w_{ij}(\text{y})=\left\{ \begin{array}{ll} \frac{1}{\int_{I_{ij}}\;d\xi }, & \;\text{if }\text{y}\in I_{ij};\\ 0, & \text{ otherwise,} \end{array}\right.$$
  or inhomogeneous in space. In this last case, it is consistent to define a probability function *w*_*ij*_ which radially decays from the actual position of the field individual *j*:
  3.18 $$w_{ij}(\text{y})=\left\{ \begin{array}{ll} \frac{R_{ij}^{2}-|\text{y}-{\text{x}}_{j}(t){|}^{2}}{\int_{I_{ij}}(R_{ij}^{2}-|\xi -{\text{x}}_{j}(t){|}^{2})\;d\xi }, & \;\text{if }\text{y}\in I_{ij};\\ 0, & \text{ otherwise.} \end{array}\right.$$
  The probabilistic interpretation of *w*_*ij*_ allows us to simulate situations in which a pedestrian is not completely sure or aware of the position of a surrounding individual. Such an uncertainty characterizes, for example, poor visibility conditions, where a walker can hear the voice of another person without seeing his/her exact location. However, it is not able to describe situations in which the walker desires to pass significantly far from another individual, as happened in our experimental test. In these cases, walker *i* psychologically overestimates the presence of an individual *j* within the region $I_{ij}$, that indeed becomes a sort of restricted/prohibited area. From a mathematical point of view, this amounts to opting for the following form of *w*_*ij*_ (see again figure 3):
  3.19 $$w_{ij}(\text{y})=\left\{ \begin{array}{ll} 1, & \text{if}\;\text{y}\in I_{ij};\\ 0, & \text{otherwise.} \end{array}\right.$$
  The function *w*_*ij*_ is the characteristic function of the region $I_{ij}$. Hereafter, it is denoted *full occupancy function*, as it models the fact that pedestrian *i* perceives the field individual *j* certainly occupying each point of $I_{ij}$.
  Regardless of the specific form given to *w*_*ij*_, hereafter named *perception function*, the repulsive interaction velocity of walker *i* resulting from a spatially distributed perception of individual *j* is defined by the following integral term:
  3.20 $${\text{v}}_{ij}^{\mathrm{rep}}(t)={\text{v}}_{ij}^{\mathrm{rep}}({\text{x}}_{i}(t),{\text{x}}_{j}(t))=\int_{\Omega }{\text{K}}_{i}(\xi -{\text{x}}_{i}(t))w_{ij}(\xi )\;d\xi$$
  where **K**_*i*_ is the already-introduced interaction kernel. In this respect, it is useful to underline that the region $I_{ij}$ constitutes the effective repulsion neighbourhood of walker *i* with respect to field individual *j*.

The overall perception-dependent repulsive term in the velocity of pedestrian *i* finally reads

3.21 $${\text{v}}_{i}^{\mathrm{rep}}(t)=\sum_{j\in S_{i}(t)}\left[(1-\lambda_{ij}(t))\;{\text{K}}_{i}({\text{x}}_{j}(t)-{\text{x}}_{i}(t))+\lambda_{ij}(t)\int_{\Omega }{\text{K}}_{i}(\xi -{\text{x}}_{i}(t))w_{ij}(\xi )\;d\xi \right],$$

where each parameter λ_*ij*_(*t*)∈{0,1} defines (for a given time *t*) how walker *i* actually perceives and therefore interacts with field individual *j*. Specifically, λ_*ij*_=0 gives a standard localized perception, whereas λ_*ij*_=1 results in a spatially distributed one. In particular, if λ_*ij*_=0 for all *j*, then the model in equation (3.4) reduces to a traditional Helbing-like fully discrete approach for pedestrian dynamics in the drag-dominated regime. In this case, there is no difference between the actual position of the field individuals and their spatial distribution as filtered by the perception of walker *i*. If, conversely, there is at least a *j* such that λ_*ij*_=1, pedestrian *i* is assumed to simultaneously consider some individuals as localized obstacles and the others as distributed entities: equation (3.4) results indeed in an integro-differential *hybrid* model. The terminology ‘hybrid’ is used because we have the coupling of both types of perceptions and relative mathematical structures. Lastly, if λ_*ij*_=1 for all *j*, pedestrian *i* perceives the rest of the crowd as a spatially extended ensemble, whose distribution is given by the superposition of the functions *w*_*ij*_, corresponding to each field individual *j*. However, also in this case, the model in equation (3.4) has a *hybrid* nature, as ${\text{v}}_{i}^{\mathrm{rep}}$ actually implements the influence that a distributed ensemble of individuals has on the dynamics of a pedestrian who is still individually represented as a pointwise/discrete element. It is useful to remark that a pedestrian may perceive and consequently approach each field individual differently. From another viewpoint, each individual can be differently perceived by the other walkers. Furthermore, as both psychological and/or environmental conditions may change, the parameters λ_*ij*_ can evolve in time, according to proper phenomenological rules.

Finally, as plotted in figure 2*c*, the interaction kernel ${\text{K}}_{i}:R^{2}\mapsto R^{2}$, which characterizes the repulsive behaviour of each moving walker *i* regardless of his/her type of perception, is given by:

3.22 $${\text{K}}_{i}(\text{z})=\left\{ \begin{array}{ll} -\frac{E_{i}}{R_{b}}\exp\left(\frac{R_{b}}{F_{i}}\right)\text{z}, & \text{if }|\text{z}|\leq R_{b};\\ -E_{i}\exp\left(\frac{2R_{b}-|\text{z}|}{F_{i}}\right)\frac{\text{z}}{|\text{z}|}, & \text{otherwise.} \end{array}\right.$$

In particular, *R*_b_ is the mean body radius already defined in (3.9), whereas *E*_*i*_ and *F*_*i*_ are constant coefficients, fixed for any individual *i*∈{1,…,*N*} and empirically estimated in [28] to 1 m s^−1^ and 0.5 m, respectively. It is worth noticing that the repulsive kernel in (3.22) is bounded and Lipschitz continuous in $R^{2}$. Analogous interaction functions, i.e. characterized by a distance-decaying behaviour at sufficiently high values of interpersonal distance, have been proposed in several well-consolidated approaches, such as in [28,36,37,45].

#### 3.1.4. Velocity random fluctuations

The dynamics of a pedestrian *i* can be also characterized by random fluctuations. In this respect, it is consistent to assume

3.23 $$\chi_{i}(t)={\bar{v}}_{i}(\cos(\chi_{i}(t)),\sin(\chi_{i}(t)))\;\forall \;i=1,\ldots ,24,$$

where *χ*_*i*_ is a uniformly distributed random angular variable, whose values fall within the range [0,2*π*] rad, and ${\bar{v}}_{i}$ is the already-introduced individual comfort speed.

### 3.2. Evolution of pedestrian gazing direction

To fully describe the behaviour of a walker *i*, the model in equation (3.4) has to be integrated by a proper evolution law for his/her gazing direction **g**_*i*_, which affects his/her interpersonal interactions by defining his/her repulsion neighbourhood. In particular, we assume each pedestrian *i* turns his/her gaze in order to reduce head rotation with respect his/her actual direction of motion. This amounts to employing the following evolution law for his/her gazing direction **g**_*i*_:

3.24 $$\frac{d\gamma_{i}}{dt}(t)=-G_{i}({\text{v}}_{i}(t)\times {\text{g}}_{i}(t))\cdot \text{k},$$

where *γ*_*i*_ is the angle between **g**_*i*_ and the *x*-axis of the domain, i.e. ${\text{g}}_{i}(t)=(\cos\gamma_{i}(t),\sin\gamma_{i}(t))$. *G*_*i*_ is a constant coefficient, set equal to 2 rads m^−1^ for all individuals *i*∈{1,…,*N*} (as estimated in [28]) and **k** is the unit vector perpendicular to the plane of motion, i.e. to the domain *Ω* (figure 2*d*). In principle, there exists several other ingredients that can be taken into account to model the evolution of the pedestrian visual field. For instance, it is possible to assume that **g**_*i*_ is constantly directed towards the individual target destination (i.e. towards ${\text{v}}_{i}^{\mathrm{des}}$, as done in [36]). Random fluctuations of individual gaze can be taken into account as well.

## 4. Numerical results

In this section, we present some sets of numerical simulations, useful to analyse the dynamics of a pedestrian resulting from different types (objective versus subjective) of perception he/she has of his/her field individuals. In particular, we start employing our model to qualitatively reproduce the above-explained experimental test. Then, we turn to provide an analysis of pedestrian walking determinants (i.e. paths) obtained, in simple and controlled configurations, by varying the individual repulsive behaviour. The values of the model parameters are finally summarized and listed in table 1.

**Table 1.** Summary of the parameters used in numerical simulations.

parameter	description	velocity term	equation	value (units)	refs.
${\bar{v}}_{i}$	comfort speed	**v**^des^_*i*_	(3.3)	1.34 (m s^−1^)	[6,36]
*A*_*i*_	wall repulsion coefficient	**v**^wall^_*i*_	(3.9)	1.0 (m s^−1^)	[28]
*B*_*i*_	wall repulsion coefficient	**v**^wall^_*i*_	(3.9)	0.01 (m)	[28]
*L*_w_	wall repulsion threshold	**v**^wall^_*i*_,	(3.9)	1.0 (m)	estimated
*R*_b_	body radius	**v**^wall^_*i*_, **v**^rep^_*i*_, **v**^cont^_*i*_	(3.9), (3.22), (3.10)	0.25 (m)	[28,36,42,43]
*C*_*i*_	body force coefficient	**v**^cont^_*i*_,	(3.10)	25.0 (s^−1^)	[28]
*D*_*i*_	sliding friction coefficient	**v**^cont^_*i*_,	(3.10)	50.0 (s^−1^)	[28]
*E*_*i*_	repulsion coefficient	**v**^rep^_*i*_,	(3.22)	1.0 (m s^−1^)	[28]
*F*_*i*_	repulsion coefficient	**v**^rep^_*i*_,	(3.22)	0.5 (m)	[28]
*θ*_*i*_	visual angle	**v**^rep^_*i*_,	(3.13)	1.48 (rad)	[28,36,44]
*R*_*i*_	visual depth	**v**^rep^_*i*_,	(3.13)	50.0 (m)	[28,36,44]

### 4.1. Reproduction of the real-world scenario

**Figure 4.**
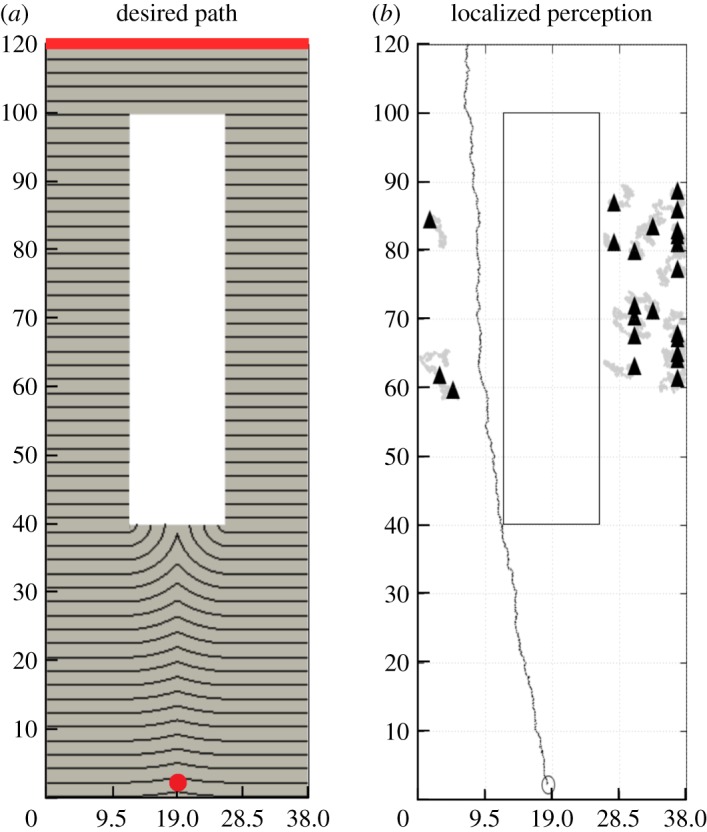
(*a*) Computational domain *Ω* and desired path of virtual pedestrian, which results in a field whose local tangent identifies the optimal direction to reach the bus stop, taking also account of a repulsive component from the domain borders and the non-walkable central flowerbed. The red line indicates the bus stop, labelled in the text by ∂*Ω*_*d*_1__, whereas the red dot indicates the initial position of all virtual pedestrians. (*b*) Representative pedestrian trajectory in the case of a localized perception of all strolling individuals, i.e. in equation (3.21), λ_1*j*_=0 for *j*=2,…,24 and for all *t*≥0.

The above-described experimental setting is computationally reproduced as follows. First, the pedestrian area of interest is reported on a numerical domain *Ω*, accounting for its geometry and dimensions (figure 4*a*). In particular, the bus stop occupies the entire upper edge of the domain. For each realization, the virtual test pedestrian (namely, *i*=1) is initially placed in **x**_1_(0)=(18.42;1.5) m, i.e. in the opposite part of the flowerbed with respect to the bus stop and exactly in the middle of the horizontal axis of the domain, so that no biases are introduced. When present, the strolling individuals are distributed according to the experimental configurations S2: three within the left lane (i.e. namely *i*=2,3,4) and 20 in the right region (namely, *i*=5,…,24). Their specific initial position is randomly assigned and maintained for each simulation run. To realistically reproduce the experimental dynamics, the simulated crowd of pedestrians is set to move according to equation (3.4), with the following velocity contributions:

4.1 $$\begin{array}{rl} & {\text{v}}_{1}(t)=\underset{{\text{v}}_{1}^{\mathrm{des}}}{\underbrace{{\text{v}}_{1}^{\mathrm{targ}}(t)+{\text{v}}_{1}^{\mathrm{wall}}(t)}}+{\text{v}}_{1}^{\mathrm{cont}}(t)+{\text{v}}_{1}^{\mathrm{rep}}(t)+\chi_{1}(t);\\ \;\text{and}\; & {\text{v}}_{i}(t)={\text{v}}_{i}^{\mathrm{wall}}(t)+{\text{v}}_{i}^{\mathrm{cont}}(t)+{\text{v}}_{i}^{\mathrm{rep}}(t)+\chi_{i}(t), \end{array}\;i=2,\ldots ,24.\}$$

The desired velocity of walker 1 **v**^des^_1_ results in a field whose local tangent identifies the optimal direction to reach the bus stop, while taking into account of the presence of the domain structural elements, i.e. the borders and the non-walkable central flowerbed. In particular, as shown in figure 4*a*, **v**^targ^_1_ is defined as in equations (3.6) and (3.7), where ∂*Ω*_*d*_1__ is here the upper boundary of the domain, i.e. the location of the target destination. A velocity contribution **v**^wall^ is also present for the pedestrians moving within the lanes around the flowerbed, who, however, do not have a desired destination but rather are strolling around their surrounding (i.e. **v**^targ^_*i*_=0 for *i*=2,…,24).

For all individuals *i*=1,…,24, the perception-dependent velocity term **v**^rep^_*i*_ is defined as in equation (3.21), with the corresponding kernel equal to (3.22), also for the set of parameter values. In particular, in all realizations, the individuals strolling around within the lanes perceive any other person as a localized entity, i.e. λ_*ij*_=0 for *i*∈{2,…,24} and *j*∈{1,…,24}. Conversely, the type of perception of walker 1 varies in the different simulation settings, as explained later on. However, the interaction set $S_{i}$ of each individual is characterized by the same extension and the gazing direction of all pedestrians evolves following equation (3.24), with the same parameter estimate.

**Figure 5.**
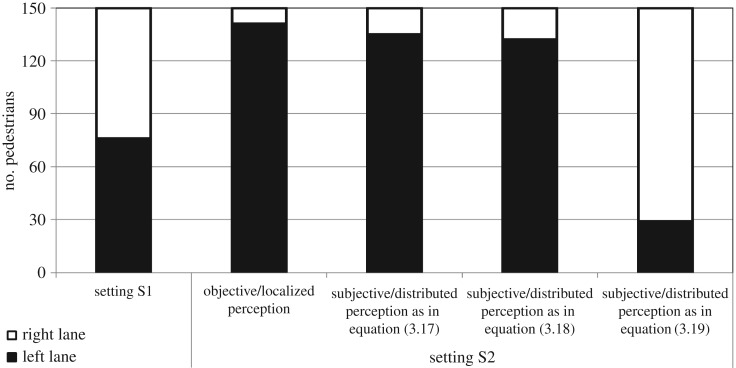
Plot summarizing the simulation results. In the case of absence of field individuals (i.e. setting S1), the virtual pedestrian equally opts for each of the two lanes, in agreement with the corresponding experimental outcomes. When the groups of individuals strolling around are added within the areas around the flowerbed, the behaviour of the sample pedestrian is instead significantly affected by his/her type of perception. If he/she objectively considers all field individuals equally and objectively, i.e. as pointwise entities, he/she opts to use the less crowded lane on the left, as in the case of experimental setting S2(a). Finally, the empirical observations relative to the experimental settings S2(b,c) cannot be computationally reproduced with the use of a localized perception but with the use of a subjective distributed perception defined by the function *w* given in equation (3.19), i.e. which models the fact that the walker psychologically overestimates the presence of the field individuals.

To match the corresponding real-world configuration, 150 numerical realizations are then run for each simulation setting. As reproduced in figure 5, in the absence of any other individual (i.e. the repulsive velocity contribution is null), the simulated test walker 1 has the same probability to take either the left or the right lane. This is consistent with the dynamics of the corresponding experimental setting S1 (cf. the plot in figure 1*c*).

When the groups of strolling individuals are added within the areas around the flowerbed, the behaviour of the sample pedestrian is instead significantly affected by his/her type of perception. In particular, if he/she considers all field individuals equally and objectively, i.e. as pointwise entities (i.e. λ_1*j*_=0 for all *j*=2,…,24), he/she opts to use the less crowded lane on the left, in agreement with the results obtained in the experimental setting S2(a) (see figures 5 and 4*b* and compare to figure 1*c*).

Walker 1 is then assumed to have the same subjective and spatially distributed perception of the individuals within the left lane, whereas those on the right remain considered as localized obstacles. In this respect, for *j*=2,3,4, we fix *R*_1*j*_=*R*_w_=1.5 m, which is a reasonable value for the size of a repulsive interaction neighbourhood, as commented in [28,42]. For *j*∈{2,3,4}, *w*_1*j*_=*w* instead take the different forms defined in the previous section.

**Figure 6.**
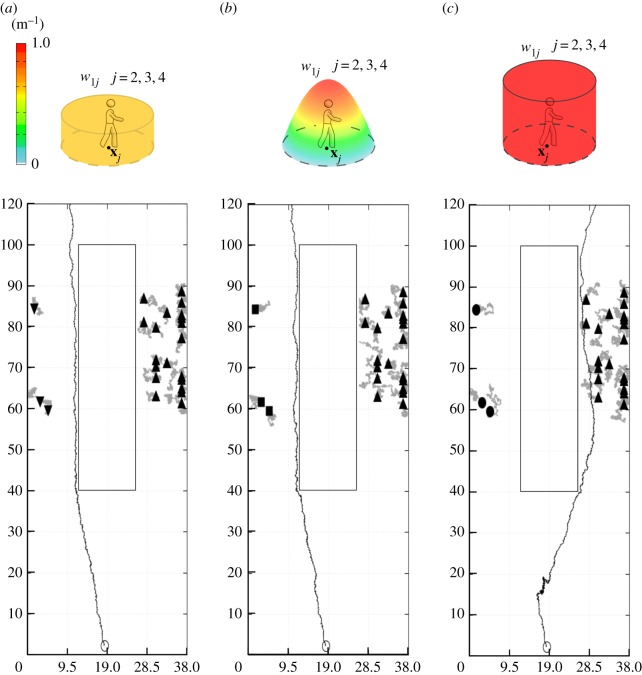
Pedestrian behaviour in the case of selected distributed perceptions of the three individuals strolling within the left lane, i.e. in equation (3.21), λ_1*j*_=1 for *j*=2,3,4 and for all *t*≥0. In particular, (*a*) reports a walker representative path resulting from *w*_1*j*_=*w* (*j*=2,3,4) as defined in equation (3.17), (*b*) reports a pedestrian representative trajectory resulting from *w*_1*j*_=*w* (*j*=2,3,4) as defined in equation (3.18), while (*c*) finally reports a walker representative path resulting from *w*_1*j*_=*w* (*j*=2,3,4) as defined in equation (3.19). In all cases, area of influence of each individual within the left lane is defined in equation (3.16), with *R*_1*j*_=*R*_w_=1.5 m for *j*=2,3,4.

As reproduced in figures 5 and 6, the empirical observations relative to the experimental settings S2(b,c) are not computationally reproduced when *w* is a probability distribution (i.e. when *w* is given by equation (3.17) or by equation (3.18)): in these cases, the walking individual still in fact prefers to pass within the left lane. This result is consistent with the fact that, as already stated, such a type of perception function models only the uncertainty that a pedestrian has of the position of his/her surrounding individuals, but does not imply psychological arguments.

Such experimental outcomes are indeed replicated only by employing the full occupancy function given in equation (3.19), which models a psychological overestimation of the presence of the field individuals within the left lane. A dramatically enhanced (and not justified from a logical viewpoint) territorial effect in fact forces walker 1 to opt for the right lane, even if it is overcrowded. In this respect, the computational results are in a remarkable agreement with the rationale underlying the experimental pedestrian behaviour. This allows us to claim that traditional purely discrete Helbing-like models of crowd dynamics are not satisfactory when approaching situations characterized by a psychological component. Such conditions can be instead more realistically reproduced by a hybrid approach, where the pedestrian repulsive behaviour is defined by a proper spatially distributed function, which is able to describe subjective perceptions of the surrounding environment.

### 4.2. Analysis of pedestrian paths

Having provided the consistency of our approach to reproduce experimental scenarios, we next turn to analyse in more detail selected pedestrian migratory determinants.

#### 4.2.1. A pedestrian and two static individuals

**Figure 7.**
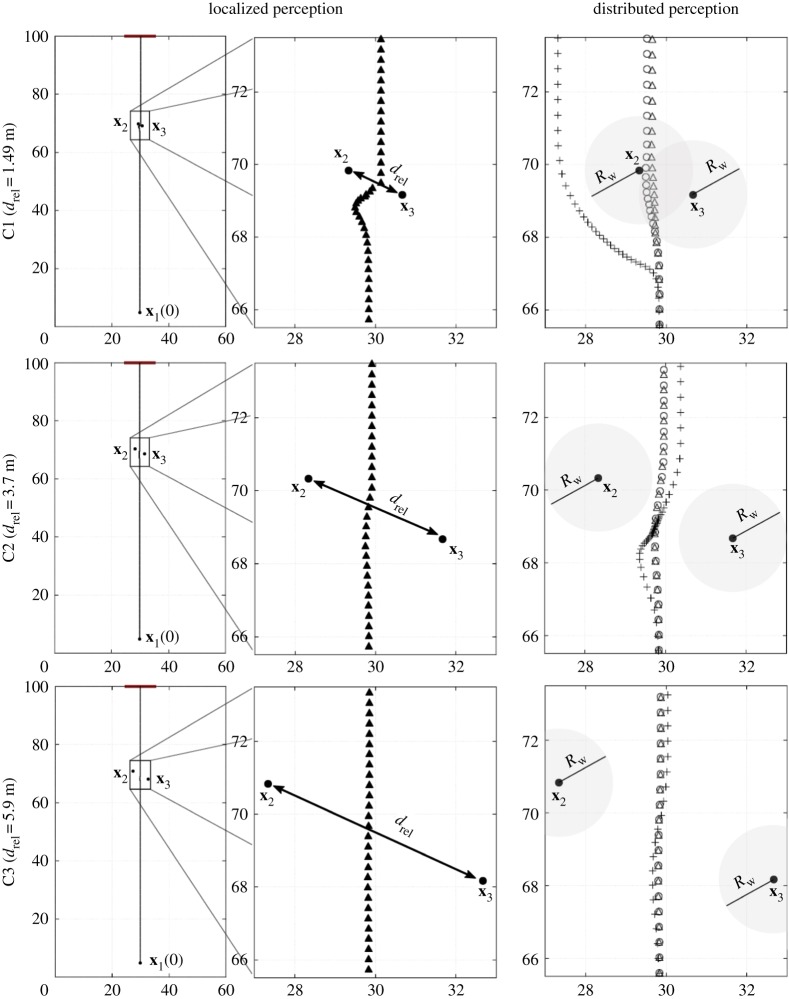
Paths of pedestrian 1 resulting from different types of perception of a pair of static individuals, placed between his/her initial position and his/her target destination. The relative distance of the static individuals varies in the different system configurations. Left column: Pedestrian trajectory in the case of an objective localized perception, i.e. in equation (3.21), λ_1*j*_=0 for *j*=2,3 and for all *t*≥0. Middle column: Blow-up images of the pedestrian behaviour when he/she is sufficiently close to the field individuals. Right column: Pedestrian trajectories in the case of subjective distributed perceptions, i.e. in equation (3.21), λ_1*j*_=1 for *j*=2,3 and for all *t*≥0. In particular, in each panel we report the paths resulting from *w*_1*j*_=*w* (for *j*=2,3) as defined either in equation (3.17) (open triangles), in equation (3.18) (open circles), or in equation (3.19) (plus symbols). In all cases, the grey shadow around each static individual represents the corresponding area of influence, defined in equation (3.16), with *R*_1*j*_=*R*_w_=1.5 m for *j*=2,3.

In this respect, in the first set of simulations, we analyse the dynamics of a pedestrian resulting from variations both in his/her type of perception and in the spatial distribution of the surrounding individuals. We indeed focus on the behaviour of a single walker *i*=1 in the presence of a pair of static individuals, i.e. in equation (3.4) we set **v**_*i*_≡0 for *i*=2,3 and for all *t*≥0. As reproduced in figure 7, pedestrian 1 is placed within a rectangular domain *Ω* of size 60×100 m: he/she wants to reach a 10-m wide target destination located at the centre of the top border of *Ω* (i.e. a large exit door, labelled by ∂*Ω*_*d*_1__). In more detail, the moving individual 1 is set to start walking from location **x**_1_(0)=(49.83;4.83) m, with initial gazing direction **g**_1_(0)=(0;1), i.e. aligned to the target destination (figure 7). In this respect, his/her desired path, defined by ${\text{v}}_{1}^{\mathrm{des}}$, consists of a straight line connecting his/her initial position **x**_1_(0) to the centre of the target door (which is also at a sufficient distance from the domain borders). However, during motion, he/she further has to avoid the static individuals, who are situated between his/her initial position and his/her target destination. The spatial distribution of the two static individuals is instead arranged according to the following configurations:

C1: **x**_2_=(49.33;69.83) m and **x**_3_=(50.67;69.1) m, with *d*_rel_=1.49 m;
C2: **x**_2_=(48.33;70.33) m and **x**_3_=(51.67;68.67) m, with *d*_rel_=3.7 m;
C3: **x**_2_=(47.33;70.83) m and **x**_3_=(52.67;68.17) m, with *d*_rel_=5.9 m,

where *d*_rel_=|**x**_2_−**x**_3_| is their relative distance, as reproduced in figure 7. It is useful to notice that, in all settings, the walker initially perceives both the static individuals, i.e. $S_{1}(0)=\{2,3\}$. The perception-dependent term ${\text{v}}_{1}^{\mathrm{rep}}$, defined in equation (3.21), then results from the superposition of the contributions arising from binary repulsive interactions between the moving walker 1 and the static field individuals falling within his/her interaction set $S_{1}$ (in the absence of domain structural elements no further restriction has to be introduced). In each configuration of the system, we then compare pedestrian behaviour upon variations in his/her type of perception of the position of the two static individuals. In this respect, in the following cases, we account for

— an objective localized perception, i.e. in equation (3.21), λ_1*j*_(*t*)≡0 for *j*=2,3 and for all *t*≥0;
— selected types of subjective distributed perception, i.e. in equation (3.21), λ_1*j*_(*t*)≡1 for *j*=2,3 and for all *t*≥0. In particular, we assume that both field individuals are equally perceived by walker 1, i.e. *w*_1*j*_=*w* for *j*∈{2,3}. Furthermore, *R*_1*j*_=*R*_w_, for *j*=2,3, is fixed again equal to 1.5 m. The perception function *w* is instead changed in the different sets of simulations, as it can assume the form of equation (3.17), (3.18) or (3.19).

It is useful to remark that we are assuming that pedestrian 1 equally perceives and approaches any static individual falling within his/her interaction set. Furthermore, his/her perception processes are not time-evolving nor affected by variable environmental conditions.

To allow a simple and immediate comparison between the dynamics generated by different types of perception, we here assume that walker motion is neither affected by physical interactions with the static crowd nor by random fluctuations (i.e. ${\text{v}}_{1}^{\mathrm{cont}}(t)=\chi_{1}(t)=0$ for all *t* in equation (3.4)). Pedestrian deviations from his/her desired strategy therefore arise only from his/her perception-dependent repulsive behaviour.

As reproduced in figure 7 (left panels), a localized perception results in the fact that the moving pedestrian passes in between the static individuals, regardless of their position. By considering them objectively as they are, i.e. as pointwise entities, walker 1 is in fact aware that their relative distance is sufficient to allow his/her passage (i.e. *d*_rel_>2*R*_b_ for all configurations). The specific position of the static individuals only and barely affects the path of pedestrian 1: the more their relative distance increases, the less pedestrian 1 deflects from the desired/optimal trajectory.

In the case of subjective distributed perceptions of the static individuals, we have that the walker passes in between them for sufficiently high *d*_rel_, i.e. =3.7 m,5.9 m, regardless of the specific form of the function *w* (=*w*_1*j*_ with *j*=2,3) (see figure 7 (right panels)). However, when *w* is given by equation (3.19), the path of pedestrian 1 visibly differs from his/her desired strategy. The underlying reason is that all types of subjective distributed perception imply that walker 1 accounts for the presence of individuals 2 and 3 over the entire areas $I_{12}$ and $I_{13}$, respectively. However, when *w* assumes the form of equations (3.17) and (3.18), the local probability to encounter one of the static individuals, as filtered by the perception of pedestrian 1, is sufficiently low (as the resulting repulsive term). The walker is indeed confident to pass within the regions $I_{12}$ and $I_{13}$ even if he/she is not completely sure of the position of the couple of static field individuals. In contrast, when *w* is given by the full occupancy function in equation (3.19), pedestrian 1 psychologically overestimates the presence of the static individuals within the areas $I_{12}$ and $I_{13}$ and therefore he/she only walks along the free space between $I_{12}$ and $I_{13}$, thereby deviating more from the optimal trajectory.

The same reasons underlie the dramatic difference in the pedestrian behaviour when the two static individuals are close to one another (i.e. when *d*_rel_=1.49 m, cf. figure 7, top-left panel). The subjective overestimation of their presence given by *w* as in equation (3.19) results in the fact that walker 1 perceives the pair of field individuals as a unique compact mass over the area $I_{12}\cup I_{13}$, that has to be avoided and circumnavigated to ‘safely’ reach the target destination.

#### 4.2.2. A pedestrian and a single static individual

**Figure 8.**
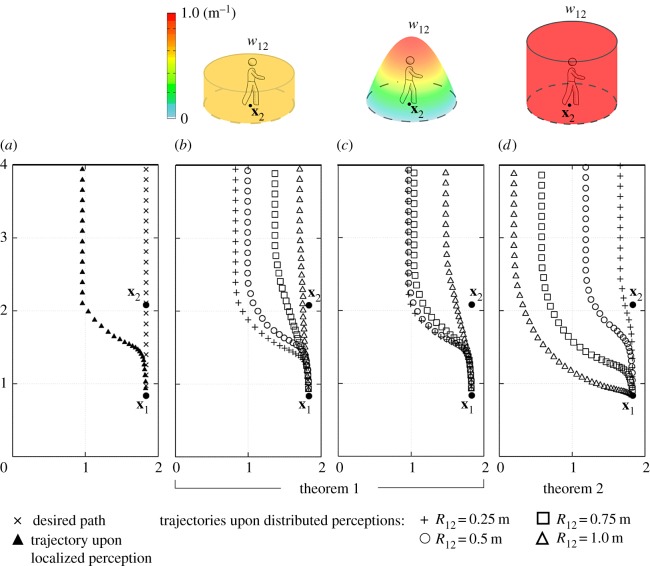
Analysis of the paths of pedestrian 1 in the presence of a single static individual 2. Panel (*a*) desired pedestrian path (cross symbols) and pedestrian trajectory in case of a localized perception of individual 2 (filled triangles). Panels (*b*,*c*,*d*) pedestrian paths resulting from a distributed perception of individual 2 as given either by equation (3.17) (*b*), by equation (3.18) (*c*) or by equation (3.19) (*d*). In each image, we plot the trajectory of pedestrian 1 in the cases of *R*_12_ equal to 0.25 m (plus symbols), 0.5 m (open circles), 0.75 m (open squares), and 1 m (open triangles).

We finally analyse how, in the case of subjective distributed perceptions, the radius determining the extension of the repulsive region affects individual behaviour. In this respect, a pair of individuals, namely 1 and 2, are placed within an 8 m^2^ domain *Ω*, which may represent the planimetry of a room or of a corridor (figure 8). Pedestrian 1 is initially located at **x**_1_(0)=(1.83;0.83) m and moves according to equation (3.4). In particular, he/she wants to reach the upper edge of the domain, i.e. ${\text{v}}_{1}^{\mathrm{targ}}(t)={\bar{v}}_{1}(0,1)$ for any time *t*≥0 (cf. figure 8*a*). During motion, he/she has to avoid individual 2, who is static for the sake of simplicity and situated at **x**_2_(*t*)=**x**_2_(0)=(1.83;2.08) m for all *t*, i.e. between the initial position of walker 1 and his/her target destination. The repulsive behaviour of pedestrian 1 is defined by equation (3.21) with the interaction kernel given in equation (3.22). It is also assumed that no other velocity contribution enters the picture (i.e. ${\text{v}}_{1}^{\mathrm{wall}}(t)={\text{v}}_{1}^{\mathrm{cont}}(t)=\chi_{1}(t)=0$ for all *t*≥0). Finally, the gaze of pedestrian 1 extends over the entire domain, so that individual 2 constantly falls within his/her interaction set, i.e. $S_{1}(t)=\{2\}$ for all *t*≥0.

When *w*_12_ has the form of a spatial probability distribution (i.e. it is given either by equation (3.17) (figure 8*b*) or by equation (3.18) (figure 8*c*)), we observe that decrements in *R*_12_ correspond to increments in the distance between the trajectory of pedestrian 1 and the position of the field individual 2. In particular, when *R*_12_ is sufficiently high, the trajectory of the moving walker is close to his/her desired strategy. In fact, by extending *R*_12_, the area $I_{12}$ in which pedestrian 1 perceives the presence of individual 2 increases but the probability of an encounter (as filtered by the subjectivity of the walker him/herself) decreases: therefore, the territorial effect is mitigated and the resulting pedestrian dynamics are mainly determined by the desired velocity component. In contrast, when *R*_12_ collapses, the behaviour of the pedestrian well approximates the one that would result from an objective localized perception of the field individual. Such a last simulation outcome is consistent with the interpretation given to equations (3.17) and (3.18): the reduction of *R*_12_ implies in fact a reduction of $I_{12}$ and therefore a reduction of the uncertainty that walker 1 has of the position of static individual 2: this finally leads to a more objective repulsive behaviour.

In the case of the full occupancy function *w*_12_ defined by equation (3.19), we have instead the inverse phenomenology: low *R*_12_ corresponds in fact to pedestrian trajectories much closer to the position of individual 2, i.e. to the desired path of walker 1 (figure 8*d*). If *R*_12_ vanishes, the psychological repulsive velocity contribution in fact disappears.

All the results proposed in this last set of simulations will be justified and formalized from a mathematical point of view in the analytical appendix of this paper.

## 5. Conclusion

In the past decades, pedestrian dynamics have been addressed by a rapidly increasing number of multidisciplinary approache that include mathematical and physical methods. Theoretical and computational tools have in fact been widely used to deal with issues concerning design, safety and security, and strategic planning for market sectors, including transport, retail, sports and the public realm. The typical models of pedestrian motion can be distinguished in macroscopic, mesoscopic and microscopic methods, according to the scale of interest. However, most of these approaches pay little attention to human perception processes, which determine how single walkers perceive and react to the presence and the position of nearby individuals.

In this respect, we have here started by presenting a real-world experiment where test walkers had to cross a pedestrian area to reach a target bus stop. In particular, they had to choose between two lanes formed by a non-walkable flowerbed in the middle of the pedestrian area. As a result, we observed that the test walkers equally used the two lanes in the absence of any other field individual. Then the less crowded region was preferentially used in the case of field individuals similar in appearance and behaviour (i.e. normally dressed students). In contrast, when a psychological component entered the picture (i.e. in the case of sloppy dressed strolling students and playing children), an unexpected phenomenology emerged: most test pedestrians in fact opted for the overcrowded lane. Taking into account such experimental outcomes, we have proposed a discrete mathematical model describing pedestrian behaviour: it consists of a first-order version of the well-known Helbing’s approach, where each simulated walker moves according to a preferred strategy influenced by physical and social interpersonal interactions, the former including collisions, the latter the desire to maintain a certain distance from the other individuals [24–25]. In particular, as a key improvement of our mathematical model with respect to the traditional version of the method, the repulsive term is here assumed to include different types of pedestrian perception of the neighbouring individuals. A walker can in fact approach the surrounding persons objectively as they are, i.e. a set of localized entities situated at their real positions, or subjectively, i.e. as elements whose presence is distributed and extended in space around their effective position. A subjective perception can account for the uncertainty that a pedestrian may have of the location and the behaviour of a surrounding individual (e.g. due to poor visibility), or it can reproduce a rationally unjustified enhancement of the territorial effect (e.g. due to fear). From a mathematical point of view, an objective perception has allowed us to maintain the purely discrete characteristics of the traditional Helbing-like approach, whereas the inclusion of subjective arguments (with the introduction of the relative continuous functions) has led to non-local integro-differential models, with hybrid characteristics. The resulting numerical outcomes have then shown that the different types of perception have the potential to greatly impact on the actual behaviour of walkers. In particular, we have observed that a traditional purely discrete model is not able to capture the phenomenology of the experimental test walkers when subjectivity plays a critical role. In this work, we have also analysed in more detail the paths of a pedestrian resulting from the different types of perception he/she has of one or of a pair of static individuals. Such simulation outcomes have been then formalized and validated with simple analytical tools.

In conclusion, it is, however, useful to remark that the inclusion of human subjectivity and psychology in a mathematical study is not trivial at all. In this respect, we do not expect that our approach is either exhaustive or definitive: rather, it is promising because, at least from a purely phenomenological/qualitative point of view, our results have been able to reproduce experimental observations. However, further analysis and considerations have to be done to find a good correspondence between possible forms of the perception function *w* and the territorial effect characterizing real-world individuals in different contexts and situations. A more quantitative validation of the model, which may, for example, derive from a comparison between empirically measured and numerical pedestrian migratory determinants, such as paths or walking times, is needed as well.

With this perspective, our work can be also considered as a first step to introduce in a realistic way the concept of environmental concern and eventually panic in a discrete model for pedestrian dynamics. This topic, out of the scope of the present paper, would involve significant modelling changes, i.e. both in determining the individual paths towards the target destination and in dealing with interpersonal interactions. As is widely known from the phenomenological literature, panicking people are in fact obsessed by short-term personal interests, uncontrolled by social and cultural constraints [40,46]. This is possibly a result of the reduced attention in situations of fear [46], which also causes options like side exits to be mostly ignored. Pedestrians entering a panic state also lose their normal repulsive behaviour, as they tend to chaotically follow other individuals, thereby clustering into more or less large groups and dropping their specific destination (especially in low-visibility situations). This phenomenology is frequently attributed to social contagion [46–49], i.e. a transition from individual to mass psychology, in which individuals transfer control over their actions to others [50], leading to conformity [51]. Such a *herding behaviour* often leads to bad overall results like dangerous overcrowding and slower escape [50,52]. The various socio-psychological theories for this contagion assume hypnotic effects, rapport, mutual excitation of a primordial instinct, circular reactions, social facilitation [53]. For these reasons, a realistic model should also include reasonable laws for the transition of each pedestrian from a normal condition to a panic state as well as rules for the corresponding contagion within the crowd.

The numerical results proposed in §(a) have also shown that our approach has the potential to be employed to describe crowd behaviour in realistic scenarios. In this respect, the proposed computational framework can be transferable to situations which involve a huge number of agents moving in more complex domains. Entering in more details, such model applications may look at the field of crowd management, particularly in relation to sports and stadia events (e.g. test of safe ingress and egress capacities), architectural projects of public and commercial buildings (e.g. optimization of internal structural elements for evacuation procedures) and designs of rail and metro stations (e.g. assessment and improvement of safety, operational integration with large-scale crowd events, evaluation of signage and communication systems). From a modelling point of view, the application of the proposed mathematical approach to one of the above-cited real-world scenarios requires the definition and the comprehensive typology of different kinds of crowds, each of them characterized by a proper perception of the surrounding groups of individuals. Possible determinants that may influence walker perception are the purpose of the moving individuals, the level of pedestrian movement (i.e. in terms of velocity and direction), the event/location atmosphere, the identification of possible crowd heterogeneities, the type of interpersonal interactions, both within the crowd and with external groups (i.e. with stewards or officers), the presence of disabilities or of luggage. Obviously, a model employment in the case of huge numbers of individuals and/or complex and large enough domains involves computational issues, i.e. mainly related to the optimization of computing time. In this respect, a possible solution is represented by the use of serial and parallel computing. High-performance serial computing can be achieved by using the same programming techniques employed in particle fluid-dynamic simulations. Otherwise, parallel computing is possible, for example, using Message Passing Interface (MPI) or shared memory parallelization. In the first case, the computational domain would be divided in subdomains that in turn would be assigned to a single processor. At each time step, each processor should communicate the pedestrians who leave its portion of domain and enter the subdomain of a neighbouring node. In case of a shared memory parallelization (e.g. on GPU devices), the computational domain and the data structure storing population data would be shared among different threads, each of them updating the state of a subset of individuals.

## Data accessibility

All the data are contained within the manuscript.

## Appendix A. Analytical results

The purpose of this section is to formalize, validate and justify from a mathematical point of view, using simple analytical methods, the pedestrian repulsive behaviour observed in the last set of simulations presented in §(b) (cf. figure 8). However, we remark that the results obtained in the following do not hold only for the cases of a walker approaching a static individual (as happened in the proposed numerical realizations) but rather they are valid also in the cases of pairs of pedestrians that both move.

**A.1. Preliminaries and formulation of the problem**

To reproduce the numerical setting, let us deal with a system formed by a pair of generic pedestrians, namely *i* and *j*, moving within a bounded two-dimensional domain $\Omega \subset R^{2}$ without structural elements. Both individuals behave according to the model defined in equation (3.4). At a given time *t*≥0, let us assume that $j\in S_{i}(t)$ and focus on the perception-dependent repulsive component of the velocity of walker *i*, which reads as

A 1 $$\begin{array}{rl} {\text{v}}_{ij}^{\mathrm{rep}}(t) & \;={\text{v}}_{ij}^{\mathrm{rep}}({\text{x}}_{i}(t),{\text{x}}_{j}(t))\\ & \;=(1-\lambda_{ij}(t))\;{\text{K}}_{i}({\text{x}}_{j}(t)-{\text{x}}_{i}(t))+\lambda_{ij}(t)\int_{\Omega }{\text{K}}_{i}(\xi -{\text{x}}_{i}(t))w_{ij}(\xi )\;d\xi . \end{array}$$

As usual, from equation (A 1), it is possible to introduce an objective localized perception by setting λ_*ij*_(*t*)=0 and a subjective distributed one with λ_*ij*_(*t*)=1. In this respect, for the sake of convenience, recalling equations (3.15) and (3.20), let us define

A 2 $$\begin{array}{rl} {\text{v}}_{ij}^{L}({\text{x}}_{i}(t),{\text{x}}_{j}(t)) & \;:={\text{K}}_{i}({\text{x}}_{j}(t)-{\text{x}}_{i}(t))\\ \;\text{and}\;{\text{v}}_{ij}^{D}({\text{x}}_{i}(t),{\text{x}}_{j}(t)) & \;:=\int_{\Omega }{\text{K}}_{i}(\xi -{\text{x}}_{i}(t))w_{ij}(\xi )\;d\xi , \end{array}\}$$

so that equation (A 1) is rewritten as

A 3 $${\text{v}}_{ij}^{\mathrm{rep}}(t)={\text{v}}_{ij}^{\mathrm{rep}}({\text{x}}_{i}(t),{\text{x}}_{j}(t))=(1-\lambda_{ij}(t))\;{\text{v}}_{ij}^{L}({\text{x}}_{j}(t),{\text{x}}_{i}(t))+\lambda_{ij}(t){\text{v}}_{ij}^{D}({\text{x}}_{j}(t),{\text{x}}_{i}(t)).$$

To provide a consistent analytical study of the repulsive velocity of pedestrian *i*, some assumptions on the interaction kernel and on the function *w*_*ij*_ (which characterizes the different types of distributed perception) have to be recalled and summarized.

Assumptions.

1. ${\text{K}}_{i}:R^{2}\mapsto R^{2}$ is a bounded and continuous field.
2. $w_{ij}:\Omega \mapsto R_{+}$ is a positive Lebesgue-integrable function, i.e. *w*_*ij*_∈*L*^1^(*Ω*), where
  A 4 $$L^{1}(\Omega )=\left\{f:\Omega \mapsto R\;s.t.\;∥f{∥}_{1}=\int_{\Omega }|f(\xi )|\;d\xi <+\infty \right\};$$
3. *w*_*ij*_ has support equal to $I_{ij}$ which, as seen, is the ball centred at the actual position of pedestrian *j* with radius *R*_*ij*_>0, i.e.
  A 5 $$\mathrm{supp}\;w_{ij}=I_{ij}=\{\xi \in \Omega :\;|\xi -{\text{x}}_{j}(t)|\leq R_{ij}\}.$$

We remark that equation (A 5) holds for any given time *t* regardless of the actual location of pedestrian *i*.

**A.2. Main results**

We are now in a position to prove the main results of this section, which establish an analytical comparison between the repulsive velocity of pedestrian *i* resulting from either a localized or a distributed perception of individual *j*, at the basis of the simulation outcomes obtained in §(b) (figure 8).

Theorem A.1 *At a given time t, let i and j be a pair of pedestrians whose actual positions* **x**_*i*_*(t) and* **x**_*j*_*(t) are such that* $j\in S_{i}(t)$*. If* **K**_*i*_ *and w*_*ij*_ *in equation (A 1) satisfy assumption 1–3 and w*_*ij*_ *is in the form of a probability distribution over* $I_{ij}$ $\left(i.e.\int_{I_{ij}}w_{ij}(\xi )\;d\xi =1\right)$ *then, when R*_*ij*_*→0 in equation (A 5), it results that
*

A 6 $$|{\text{v}}_{ij}^{L}({\text{x}}_{i}(t),{\text{x}}_{j}(t))-{\text{v}}_{ij}^{D}({\text{x}}_{i}(t),{\text{x}}_{j}(t))|⟶0,$$

Proof. From equation (A 2) and the properties of **K**_*i*_, we have that, at a given time *t*,

$$\begin{array}{rl} & \;|{\text{v}}_{ij}^{L}({\text{x}}_{i}(t),{\text{x}}_{j}(t))-{\text{v}}_{ij}^{D}({\text{x}}_{i}(t),{\text{x}}_{j}(t))|=\left|{\text{K}}_{i}({\text{x}}_{j}(t)-{\text{x}}_{i}(t))-\int_{\Omega }{\text{K}}_{i}(\xi -{\text{x}}_{i}(t))w_{ij}(\xi )\;d\xi \right|\\ & \;\;\leq \int_{\Omega }|{\text{K}}_{i}({\text{x}}_{j}(t)-{\text{x}}_{i}(t))-{\text{K}}_{i}(\xi -{\text{x}}_{i}(t))|w_{ij}(\xi )\;d\xi \\ & \;\;=\int_{I_{ij}}|{\text{K}}_{i}({\text{x}}_{j}(t)-{\text{x}}_{i}(t))-{\text{K}}_{i}(\xi -{\text{x}}_{i}(t))|w_{ij}(\xi )\;d\xi \\ & \;\;\leq \underset{\xi \in I_{ij}}{\sup}|{\text{K}}_{i}({\text{x}}_{j}(t)-{\text{x}}_{i}(t))-{\text{K}}_{i}(\xi -{\text{x}}_{i}(t))|\int_{I_{ij}}w_{ij}(\xi )\;d\xi \\ & \;\;=\underset{\xi \in I_{ij}}{\sup}|{\text{K}}_{i}({\text{x}}_{j}(t)-{\text{x}}_{i}(t))-{\text{K}}_{i}(\xi -{\text{x}}_{i}(t))|. \end{array}$$

Recalling the definition of the area of influence $I_{ij}(t)$ given in equation (A 5), we have that if $R_{ij}\to 0$ then

$$\underset{\xi \in I_{ij}}{\sup}|{\text{K}}_{i}({\text{x}}_{j}(t)-{\text{x}}_{i}(t))-{\text{K}}_{i}(\xi -{\text{x}}_{i}(t))|⟶0.$$

Therefore, equation (A 6) finally holds true. ▪

If the interaction kernel **K**_*i*_ furthermore satisfies the assumption of Lipschitz continuity, as in the case of the field employed for the simulations of the previous sections, it is possible to obtain an accurate comparison of the discrepancy between ${\text{v}}_{ij}^{L}$ and ${\text{v}}_{ij}^{D}$, at any given time *t*. In this respect, we can prove

Corollary A.2 *If* **K**_*i*_ *and* *w*_*ij*_ *in equation* (*A 1*) *satisfy assumptions 1–3 and* **K**_*i*_ *is also a Lipschitz continuous field, i.e. there exists a constant* *Lip*(**K**_*i*_)>0 *such that*

A 7 $$|{\text{K}}_{i}(\text{y})-{\text{K}}_{i}(\text{z})|\leq \mathrm{Lip}({\text{K}}_{i})|\text{y}-\text{z}|,\;\forall \;\text{y},\text{z}\in R^{2},$$

*then*

A 8 $$|{\text{v}}_{ij}^{L}({\text{x}}_{i}(t),{\text{x}}_{j}(t))-{\text{v}}_{ij}^{D}({\text{x}}_{i}(t),{\text{x}}_{j}(t))|\leq \mathrm{Lip}({\text{K}}_{i})\;\underset{\xi \in I_{ij}}{\sup}|{\text{x}}_{j}(t)-\xi |\;\forall \;t\geq 0.$$

Proof. From equations (A 2) and (A 7), we have that

A 9 $$\begin{array}{rl} |{\text{v}}_{ij}^{L}({\text{x}}_{i}(t),{\text{x}}_{j}(t))-{\text{v}}_{ij}^{D}({\text{x}}_{i}(t),{\text{x}}_{j}(t))| & \;=\left|{\text{K}}_{i}({\text{x}}_{j}(t)-{\text{x}}_{i}(t))-\int_{\Omega }{\text{K}}_{i}(\xi -{\text{x}}_{i}(t))w_{ij}(\xi )\;d\xi \right|\\ & \;\leq \int_{\Omega }|{\text{K}}_{i}({\text{x}}_{j}(t)-{\text{x}}_{i}(t))-{\text{K}}_{i}(\xi -{\text{x}}_{i}(t))|w_{ij}(\xi )\;d\xi \\ & \;\leq \mathrm{Lip}({\text{K}}_{i})\int_{\Omega }|{\text{x}}_{j}(t)-\xi |w(\xi )\;d\xi \\ & \;=\mathrm{Lip}({\text{K}}_{i})\int_{I_{ij}}|{\text{x}}_{j}(t)-\xi |w(\xi )\;d\xi \\ & \;\leq \mathrm{Lip}({\text{K}}_{i})\underset{\xi \in I_{ij}}{\sup}|{\text{x}}_{j}(t)-\xi |\int_{I_{ij}}w(\xi )\;d\xi \\ & \;=\mathrm{Lip}({\text{K}}_{i})\underset{\xi \in I_{ij}}{\sup}|{\text{x}}_{j}(t)-\xi |. \end{array}$$

 □

Corollary A.2 is indeed a particular case of theorem A.1, which holds if the interaction kernel **K**_*i*_ is a Lipschitz continuous field. In fact, if *R*_*ij*_→0, in equation (A 8) we have that $\underset{\xi \in I_{ij}}{\sup}|{\text{x}}_{j}(t)-\xi |⟶|{\text{x}}_{j}(t)-{\text{x}}_{j}(t)|=0$ and therefore equation (A 6) is recovered.

Theorem A.1, as well as the relative corollary A.2, states that the discrepancy between the repulsive behaviour of a given pedestrian *i* resulting from either an objective localized or a subjective distributed perception of a field individual *j* reduces as the extension of the area of $I_{ij}$ decreases, given a probabilistic interpretation of function *w*_*ij*_. Theorem A.1 and corollary A.2 are indeed the formalization, from a mathematical point of view, of the numerical outcomes represented in figure 8 (second and third panels).

If the function *w*_*ij*_ is a full occupancy function, which is constantly equal to 1, as defined in equation (3.19), another analytical result holds:

Theorem A.3 *At a given time t, let i and j be a pair of pedestrians whose actual positions* **x**_*i*_*(t) and* **x**_*j*_*(t) are such that* $j\in S_{i}(t)$*. Let also* **K**_*i*_ *and w*_*ij*_ *in equation (A 1) satisfy assumptions 1–3. Assume finally that w*_*ij*_*≡1 all over* $I_{ij}$*, i.e. it has the form defined in equation (3.19). If* $R_{ij}\to 0$*, it then results that
*

A 10 $$|{\text{v}}_{ij}^{D}({\text{x}}_{i}(t),{\text{x}}_{j}(t))|⟶0.$$

Proof. From equation (A 2) and the properties of **K**_*i*_, we have that at a given *t*

$$\begin{array}{rl} |{\text{v}}_{ij}^{D}({\text{x}}_{i}(t),{\text{x}}_{j}(t))| & \;=\left|\int_{\Omega }{\text{K}}_{i}(\xi -{\text{x}}_{i}(t))w(\xi )\;d\xi \right|=\left|\int_{I_{ij}}{\text{K}}_{i}(\xi -{\text{x}}_{i}(t))\;d\xi \right|\\ & \;\leq \underset{\xi \in I_{ij}}{\sup}|{\text{K}}_{i}(\xi -{\text{x}}_{i}(t))|\int_{I_{ij}}d\xi \\ & \;\leq \underset{\xi \in I_{ij}}{\sup}|{\text{K}}_{i}(\xi -{\text{x}}_{i}(t))|\;\pi R_{ij}^{2}. \end{array}$$

Recalling the definition of $I_{ij}$, it then results that if $R_{ij}\to 0$

$$|{\text{v}}_{ij}^{D}({\text{x}}_{i}(t),{\text{x}}_{j}(t))|\leq \underset{\xi \in I_{ij}}{\sup}|{\text{K}}_{i}(\xi -{\text{x}}_{i}(t))|\pi R_{ij}^{2},\to 0$$

and that therefore equation (A 10) holds true. ▪

Theorem A.3 states that if the region of influence $I_{ij}$ collapses, the corresponding repulsive contribution vanishes. In other words, when $R_{ij}\to 0$, pedestrian *i* no longer takes into account the presence of field individual *j*, even if the repulsive term is determined by a subjective perception given by the full occupancy function which is in principle set to model a psychological overestimation that *i* has of the presence of *j*. This is consistent with the results obtained from the numerical simulations summarized in figure 8*d*.
